# Assessment of myocardial repolarisation parameters in patients with familial Mediterranean fever

**DOI:** 10.5830/CVJA-2016-074

**Published:** 2017

**Authors:** Kayıhan Karaman, Metin Karayakalı, Ertuğrul Erken,, Ahmet Demirtaş, Mustafa Öztürk, Fatih Altunkaş, Arif Arısoy, Oğuzhan Ekrem Turan, Köksal Ceyhan, Ataç Çelik

**Affiliations:** Department of Cardiology, Gaziosmanpasa University School of Medicine, Tokat, Turkey; Department of Cardiology, Gaziosmanpasa University School of Medicine, Tokat, Turkey; Department of Cardiology, Gaziosmanpasa University School of Medicine, Tokat, Turkey; Department of Cardiology, Gaziosmanpasa University School of Medicine, Tokat, Turkey; Department of Cardiology, Gaziosmanpasa University School of Medicine, Tokat, Turkey; Department of Cardiology, Gaziosmanpasa University School of Medicine, Tokat, Turkey; Department of Cardiology, Gaziosmanpasa University School of Medicine, Tokat, Turkey; Department of Cardiology, Gaziosmanpasa University School of Medicine, Tokat, Turkey; Department of Cardiology, Gaziosmanpasa University School of Medicine, Tokat, Turkey; Department of Cardiology, Gaziosmanpasa University School of Medicine, Tokat, Turkey

**Keywords:** familial Mediterranean fever, myocardial repolarisation, cTp-Te interval, cTp-Te/QT ratio

## Abstract

**Background:**

Familial Mediterranean fever (FMF) is a chronic, recurrent auto-inflammatory disease characterised by self-terminating attacks of fever and sterile polyserositis. The main cause of death in auto-inflammatory diseasesis cardiovascular events. Additionally, auto-inflammatory diseases have potential effects on the myocardial repolarisation parameters, including the T-wave peak-to-end (Tp-Te) interval, cTp-Te interval (corrected Tp-Te) and the cTp-Te/ QT ratio. The aim of this study was to analyse the efficacy of myocardial repolarisation alterations in anticipation of cardiovascular risks in patients with FMF.

**Methods:**

This study included 66 patients with FMF and 58 healthy control subjects. Tp-Te and cTp-Te intervals and the cTp-Te/QT ratio were measured from the 12-lead electrocardiogram.

**Results:**

In electrocardiographic parameters, analysis of QT, QT dispersion, corrected QT (QTc) and QTc dispersion were similar between the groups. The Tp-Te and cTp-Te intervals and Tp-Te/QT and cTp-Te/QT ratios were significantly prolonged in FMF patients. Multivariate linear regression analyses indicated that erythrocyte sedimentation rate was an independent predictor of a prolonged cTp-Te interval.

**Conclusions:**

Our study revealed that when compared with control subjects, Tp-Te and cTp-Te intervals and cTp-Te/QT ratio were increased in FMF patients.

## Background

Familial Mediterranean fever (FMF) is a chronic, recurrent autoinflammatory disorder with autosomal recessive inheritance characterised by self-limiting attacks of arthritis, peritonitis or pleuritis and fever.^1^ Around the Mediterranean it is a common disease and most commonly occurs in Jews, Turks, Armenians and Arabs.^2^ FMF could become a major public health issue in these regions; therefore, early diagnosis and the necessary treatment are very important to the patient’s prognosis.

Mutations in the MEFV (MEditerranean FeVer) gene, which encodes for a protein called pyrin, is necessary for a diagnosis of FMF.^3^ Mutated pyrin is associated with uncontrolled inflammation and it increases circulatory levels of acute-phase reactants and cytokines secreted by the neutrophils.^4^ It has been shown that underlying subclinical inflammation is present during attack-free periods in patients with FMF.^3^

Chronic systemic inflammation accelerates the natural process of atherosclerosis. The main cause of death in auto-inflammatory disorders is related to atherosclerosis and cardiovascular events.^5^ Several studies have shown that the cardiovascular effects of systemic inflammation may include increased frequency of lifethreatening ventricular arrhythmias,^6^ conduction disturbances,^7^ and cardiac autonomic dysfunction^8^ in auto-inflammatory disorders.

The T wave on electrocardiography (ECG) indicates myocardial repolarisation, which can be evaluated with several ECG parameters, such as QT interval (QT), QT dispersion (QTd), corrected QT interval (QTc) and transmural dispersion of repolarisation.^9^,^10^ These parameters are usually used to diagnose pathology and detect risk of life-threatening ventricular arrhythmias. The T_peak_–T_end_ interval (Tp-Te), which is the interval between the peak and the end-point of the T wave on a resting ECG, and Tp-Te/QT ratio are accepted as a novel index of myocardial repolarisation. These are associated with an increased risk of ventricular arrhythmias.^11^

In this study, we investigated the possible effects on myocardial repolarisation of ongoing subclinical inflammation in FMF patients by analysis of ECGs. We aimed to evaluate the efficacy of the prediction of cardiovascular risks of possible non-specific repolarisation changes in patients with FMF.

## Methods

This study had a cross-sectional and observational design. Between August 2014 and January 2015, 66 FMF patients without cardiovascular involvement (39 females; mean age 28.6 ± 8.7 years), who were diagnosed with FMF according to the Tell– Hashomer diagnostic criteria,12 and 58 healthy controls matched for gender and age (35 females; mean age 28.7 ± 8.5 years), were included in this study. All the patients with FMF were treated chronically with colchicine. Mean duration of time elapsed from diagnosis of FMF was 7.9 ± 4.9 years.

Excluded from the study were patients with diabetes mellitus, hypertension, congestive heart failure, smoking, coronary artery disease, valvular heart disease, previous history of myocardial infarction, hyperthyroidism, hypothyroidism, atrial fibrillation, chronic kidney disease, chronic obstructive pulmonary disease, bundle brunch block, atrioventricular block and malignancy. The patients were evaluated with echocardiography and 12-lead ECG at least 10 days after an attack. Levels of haemoglobin and C-reactive protein (CRP), and erythrocyte sedimentation rate (ESR) and white blood cell count (WBC) were obtained from laboratory records.

The study protocol was approved by the institutional ethics committee. Informed written consent was obtained from each patient.

Twelve-lead ECGs (10 mm/mV, 25 mm/s, Cardiofax V; Nihon Kohden Corp, Tokyo, Japan) were obtained with the subject at rest in the supine position. All ECGs were transferred to a computer via a scanner and then used at 400% magnification via Adobe Photoshop software. Measurements of Tp-Te and QT intervals were performed on the computer by two experienced cardiologists, who were blinded to the clinical data of each patient and control subject.

QT and R-R intervals were measured in all derivations. The QT interval was defined as the time from the start of the QRS to the point at which the T wave returns to the isoelectric line. The R-R interval, which was measured as the average of three complexes, was used to calculate heart rate, and the QTc was calculated with Bazett’s formula.^13^ The QTd was defined as the difference between the maximum and minimum QT interval in different leads. Excluded from the study were subjects with U waves and low-amplitude T waves on their ECGs.

Although tail and tangent methods can be used in the measurement of Tp-Te interval, the tail method is a better predictor of mortality than the tangent method,^14^ and was therefore used in this study. In this method, the Tp-Te interval was defined as the interval from the peak to the end of the T wave to the point where the wave reached the isoelectric line.^15^ Measurement of the Tp-Te interval was obtained from leads V2 and V5, corrected for heart rate (cTp-Te).^11^ The Tp-Te/QT and cTp-Te/QT ratios were calculated from these measurements.

Measurements were made by two independent cardiologists taking the average of three consecutive beats. Intra-observer variability for Tp-Te interval obtained from leads V2 and V5 were 3.4 and 3.8%, respectively. Furthermore, inter-observer variability for the Tp-Te interval obtained from leads V2 and V5 were 2.5 and 2.9%, respectively.

All echocardiographic examinations (General Electric Vivid S5, Milwaukee, WI, USA) were performed in all subjects using a 2.5–3.5-MHz transducer in the left decubitus position. Two-dimensional and pulsed Doppler measurements were obtained using the criteria of the American Society of Echocardiography.^16^ Left ventricular ejection fraction (LVEF) was assessed using Simpson’s method. Left ventricular end-diastolic and end-systolic volumes (LVEDV and LVESV) were performed using Simpson’s method in the apical four- and two-chamber views at end-diastole and end-systole.

## Statistical analysis

Statistical analyses were performed using SPSS software (SPSS 18.0 for windows, Inc, Chicago, IL, USA). Categorical variables are expressed as n (%) and continuous variables are expressed as mean ± standard deviation. Mean values of continuous variables were compared between the groups using the Student’s t-test. The chi-squared test was used to assess differences between categorical variables. The relationship between parameters was determined using Pearson’s coefficient of correlation. Multivariate linear regression analysis was used to identify the independent predictors of prolonged cTp-Te interval and independent variables that differed significantly in the bivariate analyses (p < 0.1). A p-value < 0.05 was considered significant.

## Results

Baseline demographic, clinical and echocardiographic characteristics of all subjects are shown in Table 1. Age, gender, body mass index, and glucose and cholesterol levels were similar in both groups. All subjects had similar heart rates, and no significant differences were observed in blood pressure between the groups. The standard echocardiographic values were within normal limits for both groups. Additionally, erythrocyte sedimentation rate (ESR) and and C-reactive protein (CRP) levels were significantly higher in FMF patients compared with the controls.

**Table 1:** Demographic, echocardiographic and biochemical characteristics in patients with FMF and controls

**	*FMF patients (n = 66)*	*Controls (n = 58)*	*p-value*
Age (years)	26.0 ± 5.0	26.5 ± 5.5	0.616
Female, n (%)	39 (59.1)	35 (60.3)	0.887
BMI (kg/m^2^)	24.1 ± 4.3	22.7 ± 4.3	0.079
BSA (m^2^)	1.96 ± 0.17	1.63 ± 0.18	0.051
E/A ratio	1.58 ± 0.5	1.46 ± 0.44	0.182
LVEDV (ml)	92.6 ± 6.5	91.3 ± 6.4	0.253
LVESV (ml)	77.4 ± 12.8	79.6 ± 12.4	0.830
LVEF (%)	55.5 ± 3.7	54.9 ± 3.4	0.352
Glucose (mg/dl)	91.5 ± 9.1	89.6 ± 7.6	0.223
(mmol/l)	(5.05 ± 0.51)	(4.97 ± 0.42)	
Total cholesterol (mg/dl)	163.9 ± 28.6	162.1 ± 32.5	0.750
(mmol/l)	(4.25 ± 0.74)	(4.20 ± 0.84)	
LDL cholesterol (mg/dl)	103.3 ± 26.3	96.4 ± 29.1	0.164
(mmol/l)	(2.68 ± 0.68)	(2.5 ± 0.75)	
Haemoglobin (mg/dl)	14.3 ± 1.5	14.8 ± 1.2	0.099
CRP (mg/l)	6.0 ± 4.8	3.8 ± 0.9	< 0.001
ESR (mm/h)	11.4 ± 12.5	6.4 ± 5.7	0.006
NLR	2.09 ± 1.05	2.0 ± 0.68	0.577
WBC count (×109/l)	7.37 ± 1.82	7.76 ± 1.93	0.244

BMI = body mass index; BSA = body surface area; LVEDV = left ventricular end-diastolic volume; LVESV = left ventricular endsystolic volume; LVEF = left ventricular ejection fraction; LDL = low-density lipoprotein; CRP = C-reactive protein; ESR = erythrocyte sedimentation rate; NLR = neutrophil–lymphocyte ratio; WBC = white blood cell. Data are presented as mean ± SD.

**Fig. 1.**
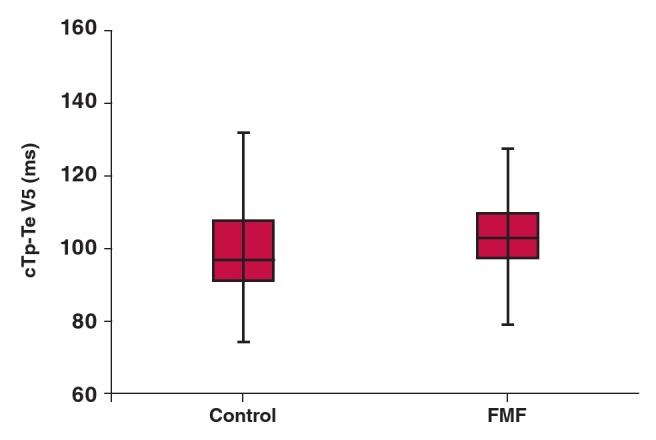
Box plot shows the cTp-Te values of FMF patients and controls.

Table 2 shows ECG measurements of the two groups. Heart rate, QT interval, QTd, QTc interval and QTc dispersion were similar between the groups. The Tp-Te and cTp-Te intervals (Fig.1), and Tp-Te/QT and cTp–Te/QT ratios were significantly prolonged in FMF patients compared to the controls.

**Table 2:** Electrocardiographic findings in patients with FMF and controls

**	**	*FMF patients (n = 66)*	*Controls (n = 58)*	*p-value*
Heart rate (beat/min)		75.3 ± 10.6	78.4 ± 12.6	0.141
QT interval (ms)	V2	351.7 ± 19.7	347.3 ± 22.8	0.256
	V5	352.3 ± 19.9	347.3 ± 22.8	0.291
QT dispersion		21.6 ± 9.4	20.7 ± 8.9	0.572
QTc interval (ms)	V2	391.0 ± 22.6	396.7 ± 31.5	0.247
	V5	391.7 ± 23.6	397.5 ± 32.2	0.252
QTc dispersion		24.0 ± 10.2	23.9 ± 10.3	0.931
Tp-Te interval (ms)	V2	97.4 ± 10.0	88.0 ± 5.7	< 0.001
	V5	93.3 ± 7.7	86.6 ± 10.2	< 0.001
cTp-Te interval (ms)	V2	108.7 ± 12.5	100.2 ± 9.7	< 0.001
	V5	104.2 ± 11.1	99.0 ± 13.9	0.023
cTp-Te/QT ratio (ms)	V2	0.31 ± 0.04	0.29 ± 0.03	0.004
	V5	0.29 ± 0.04	0.27 ± 0.03	0.009

QTc = corrected QT; Tp-Te = T-wave peak-to-end interval; cTp-Te: corrected Tp-Te. Data are presented as mean ± SD.

**Fig. 2.**
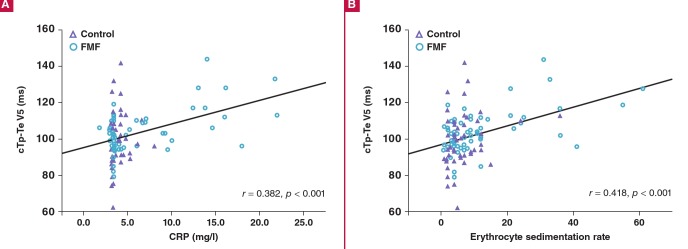
Correlations between cTp-Te interval and C-reactive protein (A), and erythrocyte sedimentation rate (B) levels.

Correlations and regression analyses between the cTp-Te interval in the V5 lead and the study parameters were performed. There were significant correlations between the cTp-Te interval and ESR (r = 0.418, p < 0.001) and CRP levels (r = 0.382, p < 0.001) and neutrophil–lymphocyte ratio (NLR) (r = 0.192, p = 0.033) and mitral E/A ratio (r = –0.190, p = 0.034) (Fig. 2A, B).

Multivariate linear regression analysis demonstrated that the ESR (β = 0.289, p = 0.004) was an independent predictor of a prolonged cTp-Te interval (Table 3). In addition there were significant correlations between the cTp-Te/QT ratio in lead V5 and ESR (r = 0.422, p <0.001) and CRP levels (r = 0.407, p < 0.001) and NLR (r = 0.207, p = 0.021) and mitral E/A ratio (r = –0.189, p = 0.035).

**Table 3:** Bivariate correlation and multivariate linear regression analyses between prolonged cTp-Te interval (lead V5) and study parameters

**	*Bivariate correlation*	*Multivariate linear regression*
*Parameters*			*r*	*p-value*	*β*	*p-value*
ESR	0.418	< 0.001	0.289	0.004
CRP	0.382	< 0.001	0.179	0.070
NLR	0.192	0.033	0.121	0.176
E/A ratio	–0.190	0.034	–0.060	0.486
Body mass index	0.165	0.067	0.074	0.387
WBC count	0.163	0.071	0.062	0.487
Age	0.063	0.484	–	–
LVEF	–0.032	0.721	–	–
Female	–0.140	0.120	–	–

CRP = C-reactive protein; ESR = erythrocyte sedimentation rate; LVEF = left ventricular ejection fraction; NLR = neutrophil–lymphocyte ratio; WBC = white blood cell.

## Discussion

In the current study, we found that cTp-Te interval and cTp-Te/ QT ratio were significantly increased in the FMF group compared to the control group. In addition we found that the increased cTp-Te interval and cTp-Te/QT ratio were positively correlated with CRP and ESR levels and NLR and mitral E/A ratios. We also found that ESR was an independent predictor of a prolonged cTp-Te interval in patients with FMF.

FMF is a hereditary transmitted auto-inflammatory disease characterised by recurrent and paroxysmal fever, peritonitis, pericarditis, arthritis and skin rashes.^1^ Some researchers have shown that subclinical inflammation continues not only during attacks, but also during the attack-free periods in patients with FMF.^17^,^18^ Ongoing, low-grade inflammation in chronic inflammatory diseases leads to deterioration of endothelial function, myocarditis, vasculitis and fibrosis of the heart muscle.^19^-^21^ It is well known that impaired endothelial function plays an important role in the pathogenesis of atherosclerosis,^22^ which has been demonstrated in FMF patients.^23^

Previous studies investigated vascular and cardiac function in FMF. Calıskan et al.^24^ analysed coronary flow reserve by transthoracic echocardiography and found that coronary microvascular function and left ventricular diastolic function were impaired in patients with FMF. This suggests that an existing inflammatory process also affects the coronary microvascular tree. In another study, Akdogan et al.^23^ showed impaired flow-mediated dilatation (FMD) of the brachial artery in patients with FMF, and impaired FMD has been shown to be correlated with coronary endothelial dysfunction.^25^ Although the exact mechanisms of arrhythmia in FMF are unknown, the authors proposed that conduction disturbances and rhythm disorders could be associated with ongoing inflammation-related ischaemia and/or focal fibrosis.

Fibrosis in the heart muscle plays an important role in the pathogenesis of ventricular arrhythmias. Parameters of autonomic cardiac tone, such as heart rate variability (HRV), heart rate turbulance (HRT) and QT dynamics, are useful for risk evaluation for ventricular arrhythmias, and abnormalities in these parameters may precede the development of fibrosis.^26^

To date, subclinical cardiovascular involvement associated with cardiac autonomic dysfunction in FMF has been reported in many studies. Fidanci et al.^27^ demonstrated that one of the time-domain parameters of HRV calculated and analysed by 24-hour ambulatory electrocardiographic monitoring software was significantly decreased in patients with FMF compared to controls. Similarly, Canpolat et al.^28^ showed abnormal HRV and HRT values in FMF patients. In light of these studies, we hypothesised that impaired endothelial function reduced coronary flow reserve and caused microvascular ischaemia and inflammation-related fibrosis, which, by affecting ventricular repolarisation, may lead to arrhythmias in FMF patients.

Myocardial repolarisation is mostly evaluated using measurements of QT interval and T wave on ECG and it may be affected by some pathophysiological processes such as genetic diseases, acquired clinical conditions, and/or drugs.^29^,^30^ Prolonged QT and QTc intervals may be caused by life-threatening ventricular arrhythmias, such as polymorphic ventricular tachycardia, torsades de pointes, and ventricular fibrillation.^31^

Several investigators have evaluated QT and QTc intervals in inflammatary diseases. In a study by Acar et al.,^32^ they reported a similar QT interval but significantly longer maximum QTc interval only in rheumatoid arthritis patients compared to control subjects. In another study, Akcay et al.^6^ showed statistically significantly longer maximum QT and maximum QTc intervals only in FMF patients. In the same study,^33^ FMF patients had similar QT and QTc intervals compared with healthy controls. In our study, QT and QTc intervals were similar between the groups.

QTd is the most frequently used parameter to detect the dispersion of ventricular repolarisation and is accepted as a marker for arrhythmia and sudden death.^34^ QTd is superior to QT and QTc intervals in the assessment of ventricular arrhythmias. It has been demonstrated that a prolonged QTd is associated with an increased risk of ventricular arrhythmias in patients with hypertrophic cardiomyopathy and long-QT syndrome.^35^,^36^ Previous studies have shown that QTd was significantly higher in some inflammatory diseases.^32^,^37^

A number of studies have investigated the effect on QTd of systemic inflammation in patients with FMF. Akcay et al.^6^ showed that QTd was increased in FMF patients. On the other hand, in another study by Giese et al.,^35^ they evaluated the QTd values in 30 FMF patients and found similar findings between FMF patients and healthy controls. We also found the QTd values were similar between the two groups.

Tp-Te interval is a new index of dispersion of myocardial repolarisation, which is related to ventricular arrhythmogenesis and sudden cardiac death.^10^,^38^ Several investigators showed that the Tp-Te interval is longer in disorders such as long-QT and Brugada syndromes.^11^ Yamaguchi et al.^39^ reported that the Tp-Te interval was more significant than QT dispersion in predicting torsade de pointes in patients with acquired long-QT syndrome.

Increased Tp-Te interval may also be a predicting index for elevated risk of cardiovascular mortality in inflammatory diseases. It was reported that the Tp-Te interval was prolonged in patients with rheumatoid arthritis and systemic lupus erythematosus.^32^,^37^ In another study, Akcay et al.^6^ reported that the Tp-Te interval was increased in FMF patients. Similar to that study, we found that the Tp-Te interval was statistically significantly prolonged in FMF patients.

Tp-Te interval is affected by variations in heart rate and body weight.15 Recently, the cTp-Te interval and cTp-Te/QT ratio were suggested to be more accurate measurements of the dispersion of myocardial repolarisation, compared to the QT, QTd, and Tp-Te intervals. In our study, we found significant differences in the cTp-Te interval and cTp-Te/QT ratio between FMF patients and control subjects. In the light of these data, we evaluated the effect of inflammatory markers on the cTp-Te interval in FMF patients and found that prolonged Tp-Te was positively correlated with ESR and CRP levels and NLR. In addition, we found that ESR was an independent predictor of a prolonged cTp-Te interval in patients with FMF.

Our study has several limitations. The major limitation is the small size of the study population and it may have negatively affected the statistical results. Second, the study had a crosssectional design and there was no follow up of arrhythmic episodes in patients.

## Conclusions

The findings of this study demonstrate that increased cTp-Te interval and cTp-Te/QT ratio may create a specific risk for ventricular arrhythmias in patients with FMF. However, the underlying mechanism and prognostic effects are as yet unknown. Therefore larger, long-term prospective and multicentre followup studies are needed.
